# The Effects of Armillarisin A on Serum IL-1β and IL-4 and in Treating Ulcerative Colitis

**DOI:** 10.1007/s12013-014-0413-7

**Published:** 2014-11-25

**Authors:** Ping Wu, Yonggao Guo, Fangyuan Jia, Xiuli Wang

**Affiliations:** Department of Gastroenterology, Xuzhou Central Hospital, Affiliated Xuzhou Hospital, Medical School of Southeast University, Xuzhou Clinical Medical College of Nanjing University of Chinese Medicine, Xuzhou, 221009 Jiangsu China

**Keywords:** Ulcerative colitis, Armillarisin, IL-1β, IL-4

## Abstract

To study the therapeutic effect of Armillarisin A on patients with ulcerative colitis (UC) and on serum IL-1β and IL-4, sixty patients with UC were randomly divided into three groups: Armillarisin A treatment group (Group I), Armillarisin-combined hormone therapy group (Group II), and hormones treatment as the control group (Group III). Patients in Group I received Armillarisin A 10 mg enema in 100 ml saline. Patients in Group II received Armillarisin A 10 mg and dexamethasone 5 mg enema in 100 ml saline. Patients in Group III received only dexamethasone 5 mg enema in 100 ml saline. The therapeutic efficacy and serum levels of IL-4 and IL-1β were observed. After 4 week treatment, the total effective rates were 90.0 % in Group I and 95.0 % in Group II. Both are higher than it in control group, which was 70.0 %. The serum levels of IL-4 in Groups I and II were significantly higher than it in control group. Compared to IL-4 levels before treatment, the levels of IL-4 after treatment were significantly higher in both Groups I and II. The serum levels of IL-11β were significantly decreased in Groups I and II in comparison to it in control group. Compared to the levels of IL-1β before treatment, the levels of IL-1β were significantly decreased. Armillarisin A shows a significant effect in treating UC. It helps increase IL-4 and lower IL-1β and the mechanism may be related to the body’s immunity regulation.

## Introduction

Ulcerative colitis (UC) is an immune-related inflammatory disease with unknown etiology. The incidence of UC is continuously increasing in China. Although the pathogenesis of UC is not yet clear, most studies suggest that it is multifactorial. In other words, the combination of internal and external factors leads to the UC. The factors consist of genetic factors, inappropriate immune response, and some certain environmental factors. In recent years, the role of cytokines in the pathogenesis, development, and prognosis of UC has been recognized and probably the imbalance between pro-inflammatory cytokines and anti-inflammatory cytokines plays a key role in the pathogenesis of UC [1, 2]. Studies have shown that interleukin (IL) plays a pivotal role during the pathogenesis of UC [3]. In this study, we investigated the efficacy of Armillarisin on IL-1β and IL-4 in UC patients.

## Materials and Methods

### Patient Information

Sixty UC patients admitted in our hospital from January 2009 to December 2012 were included in this study. Patients were randomly divided into three groups. Armillarisin treatment group (Group I) includes 20 patients. Of the 20 patients, 12 are males and 8 are females with age range from 18 to 68 years old. The average is 39.62 ± 11.24 years old. The courses of disease were among 6 months to 11 years. The mean is 4.20 ± 4.75 years. Severity grading categorizes patients to mild 14 cases and moderate 6 cases. Armillarisin-combined hormone group (Group II) includes 20 patients. Of the 20 patients, 14 are males and 6 are females with age range from 17 to 69 years old. The average is 38.42 ± 10.54 years old. The courses of disease were among 5 months and up to 10 years. The mean is 5.20 ± 4.15 years. Severity grading categorizes patients to mild 13 cases and moderate 7 cases. Control group with only hormone regiment (Group III) includes 20 patients. Of the 20 patients, 11 are males and 9 are females with age range from 19 to 70 years old. The average is 39.62 ± 11.24 years old. The courses of disease were among 5 months to 10 years. The mean is 5.15 ± 3.75 years. Severity grading categorizes patients to mild 14 cases and moderate 6 cases. The difference of gender, age, duration, and severity among three groups are not statistically significant (*P* > 0.05).

### Diagnosis Criteria

Referring to the 2007 diagnosis criteria made by the cooperative experts group of inflammatory bowel diseases, Digestive Disease Association of Chinese Medical Association [4].

### Inclusion Criteria

Patients were included if they were satisfied with all of the criteria:

- Meet the diagnostic criteria for ulcerative colitis by colonoscopy exam 1 week before enrollment;
- Clinical types are active UC patients with chronic relapsing;
- Mild-to-moderate UC patients;
- Aged 17–70 years old; and
- Signed informed consent.

### Exclusion Criteria

Patients were excluded if they have any of: (1) Severe primary diseases of cerebrovascular, liver, kidney, and hematopoietic systems. (2) Serious complications, such as partial stenosis, intestinal obstruction, intestinal perforation, rectal polyps, rectal distension toxic, colon, colorectal cancer patients, and pregnant or lactating women.

### Treatment

Patients in Group I received enema with Armillarisin 10 mg in saline 100 ml every night; Patients in Group II received enema with Armillarisin 10 mg and dexamethasone 5 mg in saline 100 ml every night; Patients in Group III received enema with dexamethasone 5 mg in saline 100 ml every night. The course of treatment was 4 weeks. All patients underwent colonoscopy before treatment and after treatment to evaluate the efficacy.

### Outcome Measurements and Detection Methods

A quantity of 5 ml fasting vein blood was taken before and after treatment for each treatment group and for the control group. The levels of serum IL-1β and IL-4 were measured using an ELISA kit (Xitang Biotechnology Co., Ltd., Shanghai, China). All procedures strictly followed the kit instruction

### Efficacy Standards [4, 5]

- *Cured* clinical symptoms vanished and the mucosa shows normal tissue by colonoscopy.
- *Significant* clinical symptoms vanished and the colonoscopy result shows that mucosa lesions are significant improved.
- *Effective* clinical symptoms vanished and the colonoscopy result shows mild inflammation of the mucosa or false mucosal polyp formation.
- *Ineffective* the clinical symptoms and the endoscopic and histological findings are not improved.

### Statistics

All data were analyzed using SPSS 16.0 software (SPSS Inc., Chicago, IL, United States). The numerical data were presented using $\bar{x} \pm s$ and tested using student *t* test. The categorical data were tested using *χ* ^2^ test. *P* *<* 0.05 indicates significant statistic difference.

## Results

### The Comparison of Efficacy Among Three Groups

After 4 weeks of treatment, the total effective rate in Group I is 90.0 %, the total effective rate in Group II is 95.0 %, and in the control group, it is 70.0 %. The differences of efficacy between Groups I and III, and between Groups II and III are statistically significant (*P* < 0.05) (Table 1).

**Table 1:** The comparison of effective rates among three groups (%)

Group	*n*	Cured		Significant effective		Effective		Ineffective		Total effective rate
		Cases	Rate	Cases	Rate	Cases	Rate	Cases	Rate	
I	20	9	45.0	6	30.0	3	15.0	2	10.0	90.0
II	20	12	60.0	6	30.0	1	5.0	1	5.0	95.0
III	20	3	15.0	5	25.0	6	30.0	6	30.0	70.0

The differences of efficacy between Groups I and III, and between groups II and III

### The Comparison of the Levels of IL-4 and IL-1β Before and After Treatment

The level of serum IL-4 was significantly increased after 4 weeks treatment, compared with the serum level before treatment. The differences of the levels of IL-4 between the treatment groups and the control group are statistically significant (*P* < 0.05) (Table 2). In comparison to the level before treatment, the level of IL-1β was significantly decreased after the treatment. The differences of the levels of IL-1β between the treatment groups and the control group are statistically significant (*P* < 0.05) (Table 3).

**Table 2:** The comparison of serum IL-4 before and after treatments among three groups ($\bar{x} \pm s$, pg/ml)

Group	*n*	Before treatment	After treatment
I	20	5.8 ± 2.1	12.2 ± 3.1**
II	20	6.1 ± 2.1	16.2 ± 4.1**
III	20	5.5 ± 4.1	8.2±2.3*

Compared to the level before treatment with group: * *P* < 0.05; Compared to Group III after treatment: ** *P* *<* 0.05

**Table 3:** The comparison of serum IL-1 before and after treatments among three groups ($\bar{x} \pm s$, ng/ml)

Group	*n*	Before treatment	After treatment
I	20	1.3 ± 0.3	0.8 ± 0.2**
II	20	1.2 ± 0.2	0.6 ± 0.3**
III	20	1.4 ± 0.3	1.0±0.2*

Compared to the level before treatment with group: * *P* < 0.05; Compared to Group III after treatment: ** *P* < 0.05

## Discussion

The main clinical manifestations of UC are diarrhea, abdominal pain, and mucus pus. UC is characteristic as long duration, broad lesions, and seriously affecting the quality of life of patients. This disease is considered to be precancerous lesions of colon cancer and has been listed as one of the modern diseases more difficult to cure by World Health Organization. The etiology and pathogenesis of UC are not entirely clear, but probably due to the autoimmune damage, heredity, infection, and neuropsychiatric factors.

In recent years, the role of cytokines in treating UC is increasing our attention. The roles of IL-1β, a pro-inflammatory cytokine, and IL-4, an anti-inflammatory cytokine, have become increasingly clear. It turns out the occurrence and development of UC probably because of the imbalance between the two cytokines. During the occurrence and development of UC, the local tissue generates a large amount of IL-1β, which activates T lymphocytes and B lymphocytes to enhance immune function. Meanwhile, IL-1β promotes the expressions of other inflammatory cytokines facilitating the neutrophil infiltration. In addition, the occurrence of UC diarrhea probably is due to the fact that IL-1β induces the release of H_2_O_2_, which impacts the release of Ca^2+^, resulting in colonic smooth muscle dysfunction in UC patients [6].

IL-4 plays a key role in maintaining intestinal immunity and the suppression and elimination of intestinal inflammation [7]. IL-4 can inhibit the production of IL-1b from monocyte-macrophage and reduce the ability of activated monocytes macrophages secreting oxygen-free radicals. Wenjie Yue and his colleagues found that the localized expression level of IL-4 in UC intestinal mucosal lesions was significantly higher than it in normal mucosa, indicating that IL-4 is involved in the pathogenesis of UC. They also found that in UC patients, the expression levels of IL-4 in severe active group patients were significantly higher than those patients in mild-to-moderate active group, suggesting that the level of IL-4 increases with the severity of the clinical disease activity increasing. Therefore, the expression level of IL-4 can be used to assess the severity of UC [8].

Armillarisin is a coumarin compound extracted from the fruiting bodies of Armillariella, or artificially synthesized. Armillarisin is mainly used for treating acute cholecystitis, chronic cholecystitis attack, other biliary tract diseases complicated by acute infection, chronic superficial gastritis, and chronic atrophic gastritis superficial [9]. Meanwhile, Armillarisin plays a role in regulating and promoting immune function and enhancing the role of macrophages. As a result, Armillarisin inhibits bacteria growth, improves the protein metabolism, and regulates liver function.

In this study, we used either Armillarisin alone or Armillarisin combined with hormone therapy in treating UC and found that after 4 weeks of treatment, the total effective rate of Armillarisin in treating UC is 90.0 %. The total effective rate of Armillarisin combined with hormone therapy is 95.0 %. Both are significantly higher than the 70.0 % total effective rate of hormone therapy alone. The serum level of anti-inflammatory cytokine IL-4 after treatment is significantly higher than it before treatment. Comparing to the levels of IL-4 after receiving hormone therapy alone, the levels of IL-4 after receiving Armillarisin along or Armillarisin combined with hormone therapy were significantly increased. The serum level of pro-inflammatory cytokine IL-1β was significantly lower in the treatment group in comparison to it in the control group. Comparing to the levels of IL-1β in control group, its level is also decreased significantly, suggesting that Armillarisin is effective in treating UC. Meanwhile, we also found that the efficacy of combined Armillarisin and hormone therapy is also better than Armillarisin alone, indicating that a combination of Armillarisin and hormone is better than either Armillarisin or hormone therapy alone in treating patients with UC.

In summary, in this study, we found Armillarisin has a significant effect intreating UC. Furthermore, the better efficacy might achieve by combining Armillarisin and hormone therapy through increasing IL-4 and decreasing IL-1β. The mechanism may be related to Armillarisin regulating the body’s immunity. This study provides a certain theoretical basis for the effective treatment of UC.

## References

[1] Sisi D, Keli D (2010). The effects of Yuyang enema regimen in treating ulcerative colitis and on serum IL-4 and IL-10. Liaoning University Traditional Chinese Medicine.

[2] Zhijie Z, Sheng X, Gaofeng M, Wenge Y, Xinshan X (2011). The effect of traditional Chinese medicine in serum IL-8 and IL-10 in ulcerative colitis patients. Shanxi Traditional Chinese Medicine.

[3] Qiangmei C (2011). The study of the mechanism of the effect of sustained release mesalamine granules on serum IL-6 and TNF-α in ulcerative colitis patients. Clinical Research.

[4] Ou, Y., Hu, P., Qian, J., Zheng, J., Hu, R.; Chinese Consensus on Diagnosis and Treatment Standard of Inflammatory Bowel Disease. Inflammatory Bowel Disease Collaborative Group, Chinese Medical Association. (2007). *Chinese Journal of Gastroenterology, 12*(8), 488–495.

[5] Hao Lijun, Tang Wenjun, Zhang Rongjuan (2011). Effect of glutamine granules enema on IFN-y, IL-4, IL-8 in patients with ulcerative colitis and observation of clinical efficacy. Chinese Journal of Nosocomiology.

[6] Cao W, Vrees MD, Potenti FM, Harnett KM, Fiocchi C, Pricolo VE (2004). Interleukin 1beta-induced production of H2O2 contributes to reduced sigmoid colonic circular smooth muscle contractility in ulcerative colitis. Journal of Pharmacology and Experimental Therapeutics.

[7] Mittal RD, Bid HK, Ghoshal UC (2005). IL-1 receptor antagonist (IL-1Ra) gene polymorphism in patients with inflammatory bowel disease in India. Scandinavian Journal of Gastroenterology.

[8] Wenjie Y, Yi L, Wei X, Le D, Xiaoting L, Weiru J, Xu S, Liang Z, Jie L (2012). The expression characteristics of IL-2, IL-4, IL-17 and IL-10 and the relationships between these cytokines and the severity of ulcerative colitis. Fudan Xuebao.

[9] Zhaomin S, Guoqiang Z (2011). The clinical observation of the effect of Armillarisin in treating acute biliary tract infection. Journal of Zhejiang Trauma Surgery.

